# Acute Necrotizing Esophagitis Following a 44-Hour Refractory Food Bolus Impaction

**DOI:** 10.7759/cureus.98036

**Published:** 2025-11-28

**Authors:** Yoshifumi Fujii, Daichi Hayashi, Miharu Kitazawa, Akira Doi, Dai Nakamatsu, Kengo Matsumoto, Masashi Yamamoto, Koji Fukui, Tsutomu Nishida

**Affiliations:** 1 Department of Gastroenterology, Toyonaka Municipal Hospital, Toyonaka, JPN

**Keywords:** acute necrotizing esophagitis, endoscopy, esophageal food impaction, food impaction, upper endoscopy

## Abstract

We report a rare case of acute necrotizing esophagitis (ANE) that developed following prolonged food bolus impaction. A 52-year-old man with a history of depression and nearly complete edentulism, but no other comorbidities, presented with acute dysphagia after ingesting a large piece of pork. Endoscopy revealed a meat bolus tightly impacted in the thoracic esophagus, with no visible lumen. Multiple endoscopic retrieval attempts using snares, retrieval nets, and dissection devices were unsuccessful due to the bolus’s large size and dense, fatty consistency. Ultimately, repeated blunt fragmentation using alligator forceps allowed dislodgment into the stomach. Endoscopic examination after removal revealed circumferential black discoloration and mucosal edema, consistent with ANE, confined to the proximal side of the obstruction. The patient was managed conservatively with bowel rest (nil per os, NPO) and intravenous fluids, and follow-up endoscopy on hospital day 7 showed marked mucosal improvement. He resumed oral intake without complications and was discharged on hospital day 9. This case highlights that prolonged refractory esophageal obstruction for 44 hours, even without systemic comorbidities, can lead to ANE through localized ischemia and mechanical stress. Prompt recognition and timely intervention are essential to prevent serious complications.

## Introduction

Food bolus impaction is one of the most common types of esophageal foreign bodies in adults, particularly in Western countries, where meat is frequently the cause [1]. The esophagus has several natural anatomical narrowings, especially at the upper esophageal sphincter, the level of the aortic arch, and the diaphragmatic hiatus, where impaction often occurs. Most cases are associated with underlying esophageal abnormalities, such as strictures, eosinophilic esophagitis (EoE), Schatzki rings, or malignancy [2]. However, food impaction can also occur in individuals without structural abnormalities, especially when a large bolus is insufficiently chewed. Additional risk factors include tooth loss, advanced age, psychiatric or neurological disorders, and chronic alcohol consumption [1]. Notably, even healthy individuals may inadvertently ingest foreign objects, especially under conditions such as hasty eating or impaired attention. Accordingly, current guidelines recommend urgent endoscopic extraction, ideally within 24 hours, as delays increase the risk of complications such as perforation and failed retrieval [1].

Acute necrotizing esophagitis (ANE), also known as "black esophagus," is a rare clinical condition characterized by circumferential mucosal necrosis and black discoloration of the esophagus [3]. Although its exact pathophysiology is not fully understood, proposed mechanisms include ischemic injury, impaired mucosal defense, and gastric acid exposure [4]. Direct evidence is limited, but prolonged esophageal impaction may reduce local perfusion via sustained mechanical compression, predisposing to focal ischemia and, in severe cases, mucosal necrosis. Clinically, ANE can lead to serious complications such as perforation and post-inflammatory stricture, and carries a nontrivial mortality; therefore, early recognition and timely management are important.

To our knowledge, few reports have linked prolonged food bolus impaction to subsequent ANE. Here, we present a rare case of ANE that developed after the delayed removal of an impacted food bolus and repeated endoscopic interventions. This case underscores a plausible association between food impaction and ANE.

## Case presentation

A 52-year-old man with a history of depression but no other comorbidities or regular medication use presented to our emergency department in late 2024 with acute dysphagia and anterior chest pain after choking on a large piece of pork during dinner. He had no prior history of dysphagia and had a long-standing habit of swallowing food without adequate mastication. Contrast-enhanced computed tomography (CT) revealed a hyperdense foreign body lodged in the thoracic esophagus. The patient was admitted for urgent endoscopic removal.

**Figure 1:**
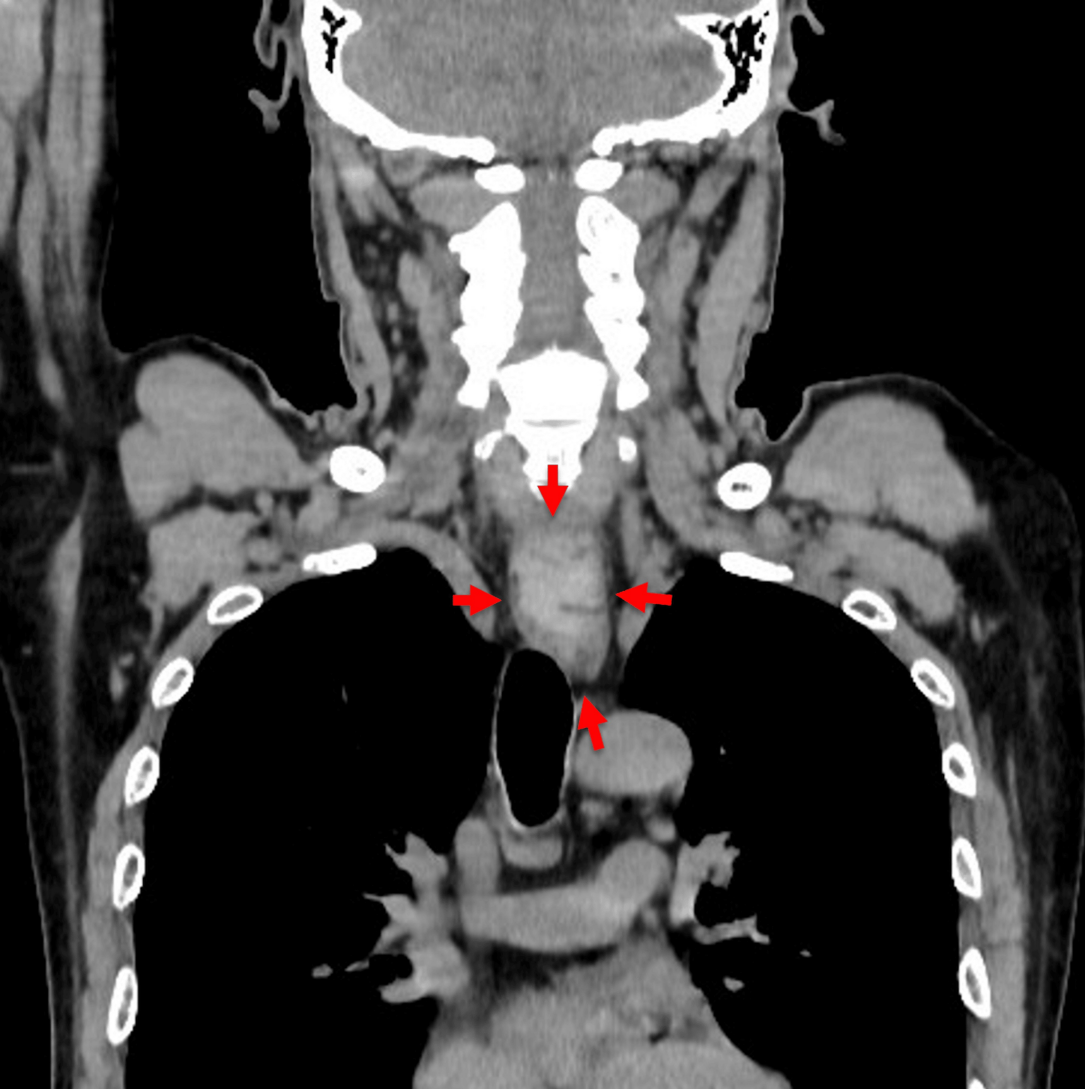
Coronal contrast-enhanced computed tomography (CT) image Coronal contrast-enhanced CT image of the chest showed a hyperdense object (arrows) lodged in the thoracic esophagus.

Upon admission, the patient’s vital signs were stable. He was 160.5 cm tall and weighed 52.4 kg. The patient was alert and oriented. Physical examination revealed that the patient was nearly edentulous and was not using dentures. He initially reported anterior chest pain; there was no subcutaneous emphysema, abnormal lung sounds, or hypoxia. There were no signs of mediastinitis or pneumonia. CT confirmed a 40 mm × 25 mm hyperdense object in the thoracic esophagus (Figure 1).

**Figure 2:**
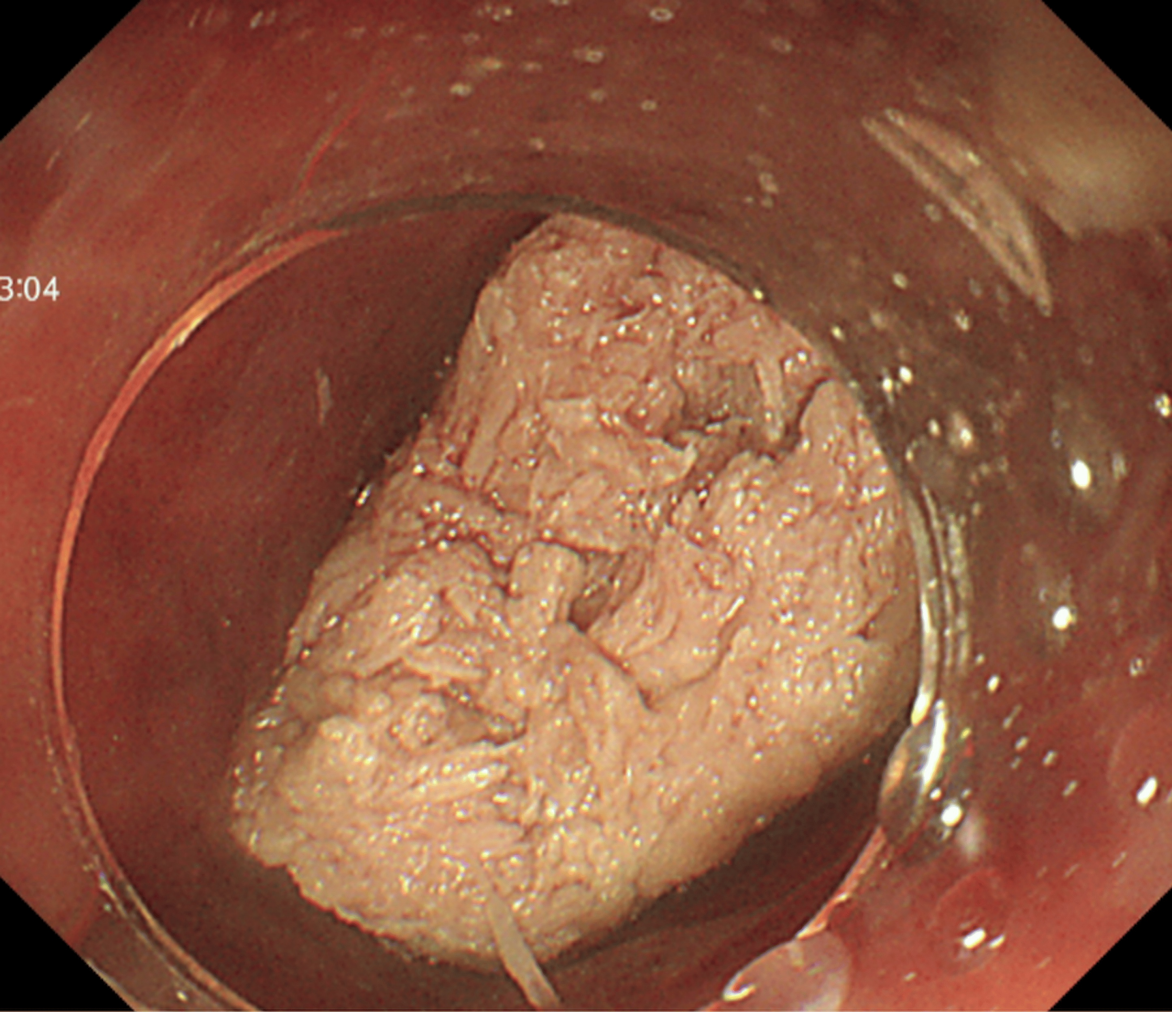
Endoscopic images Endoscopic images showed a tightly impacted meat bolus occluding the esophageal lumen with no visible gap between the bolus and the mucosa.

Emergency esophagogastroduodenoscopy (EGD) was performed on the day of admission using a GIF-Q260J (Olympus Optical, Tokyo, Japan), revealing a large piece of meat tightly impacted in the esophagus with no visible lumen around the bolus. Initial attempts to push the bolus into the stomach were unsuccessful because of its large size (Figure 2).

Retrieval using grasping forceps, a retrieval net, and a snare also failed. The patient was kept nothing by mouth and managed conservatively overnight with intravenous proton pump inhibitor therapy.

**Figure 3:**
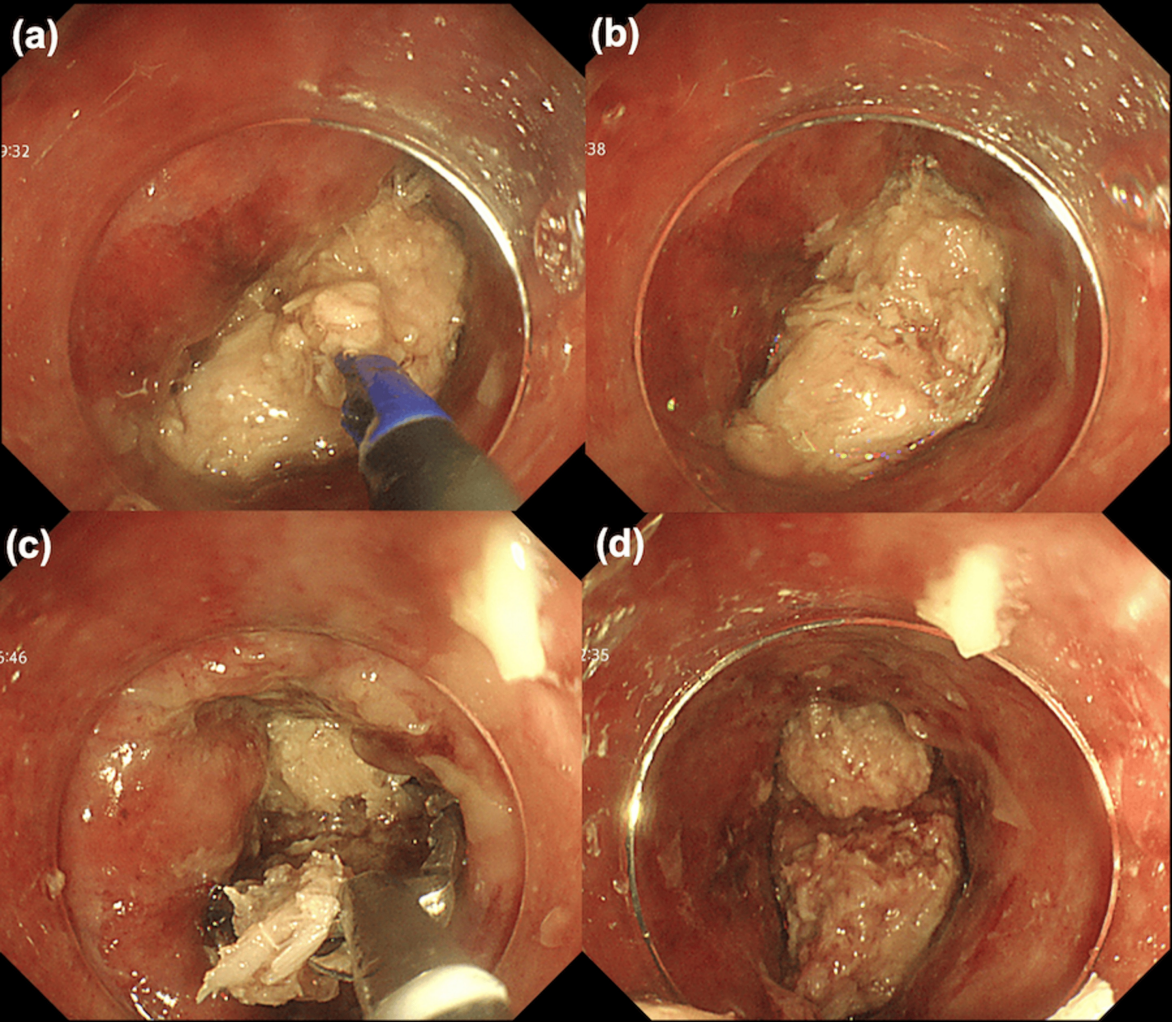
Endoscopic fragmentation of the impacted meat bolus Attempted dissection using a scissor-type knife was ineffective due to high fat content (a, b). Mechanical fragmentation using alligator forceps was partially effective but required multiple sessions (c, d).

On hospital day 2, a transnasal endoscopy (GIF-1200N; Olympus Optical, Tokyo, Japan) was attempted, but the scope could not pass because of the size of the impacted bolus. A two-channel endoscope (GIF-2TQ260M; Olympus Optical, Tokyo, Japan) was used. Two different grasping forceps were inserted through the dual channels of the endoscope to retrieve the object. We attempted to retrieve the food bolus by simultaneously grasping it with two forceps introduced through a dual-channel endoscope. A retrieval net was also employed; however, the bolus was too large to fit within it. Snaring was attempted; however, the large size of the bolus and the narrow space between it and the esophageal wall made this technique unfeasible. Dissection using a needle knife and scissor-type knife was also attempted; however, due to the high fat content of the bolus, cutting was inefficient, and it could not be reduced to a retrievable size (Figures 3a-3b). Ultimately, repeated blunt fragmentation of the bolus at its center using alligator forceps was judged to be the most effective strategy; however, the procedure was prolonged, and complete removal was deferred again (Figures 3c-3d).

**Figure 4:**
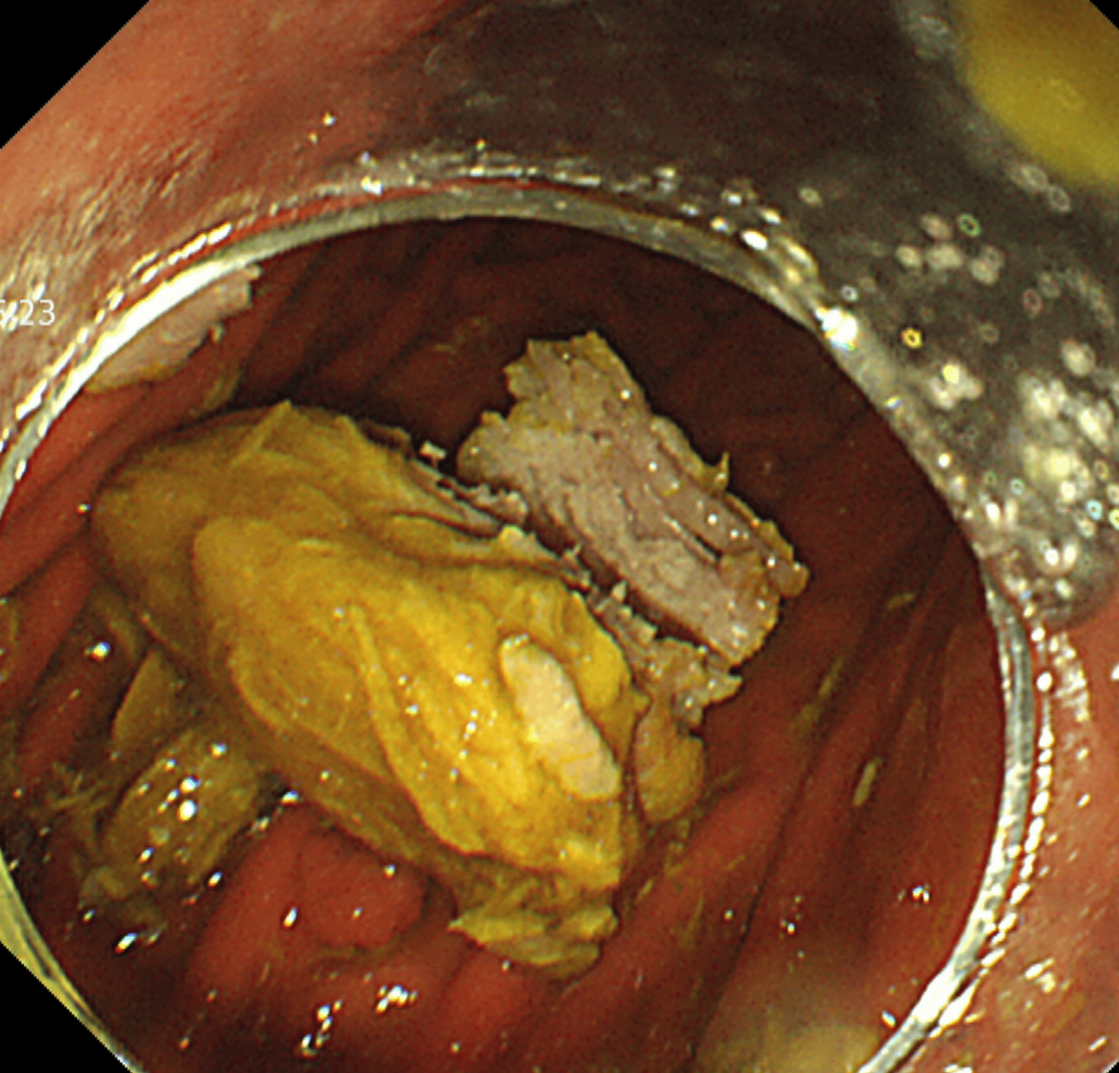
Endoscopic image The endoscopic image showed the dislodged meat bolus in the gastric lumen after successful fragmentation and advancement from the esophagus.

On hospital day 3, blunt fragmentation using alligator forceps was repeatedly performed at the center of the impacted food bolus, eventually enabling successful dislodgment and passage into the stomach using a GIF-Q260J (Figure 4).

**Figure 5:**
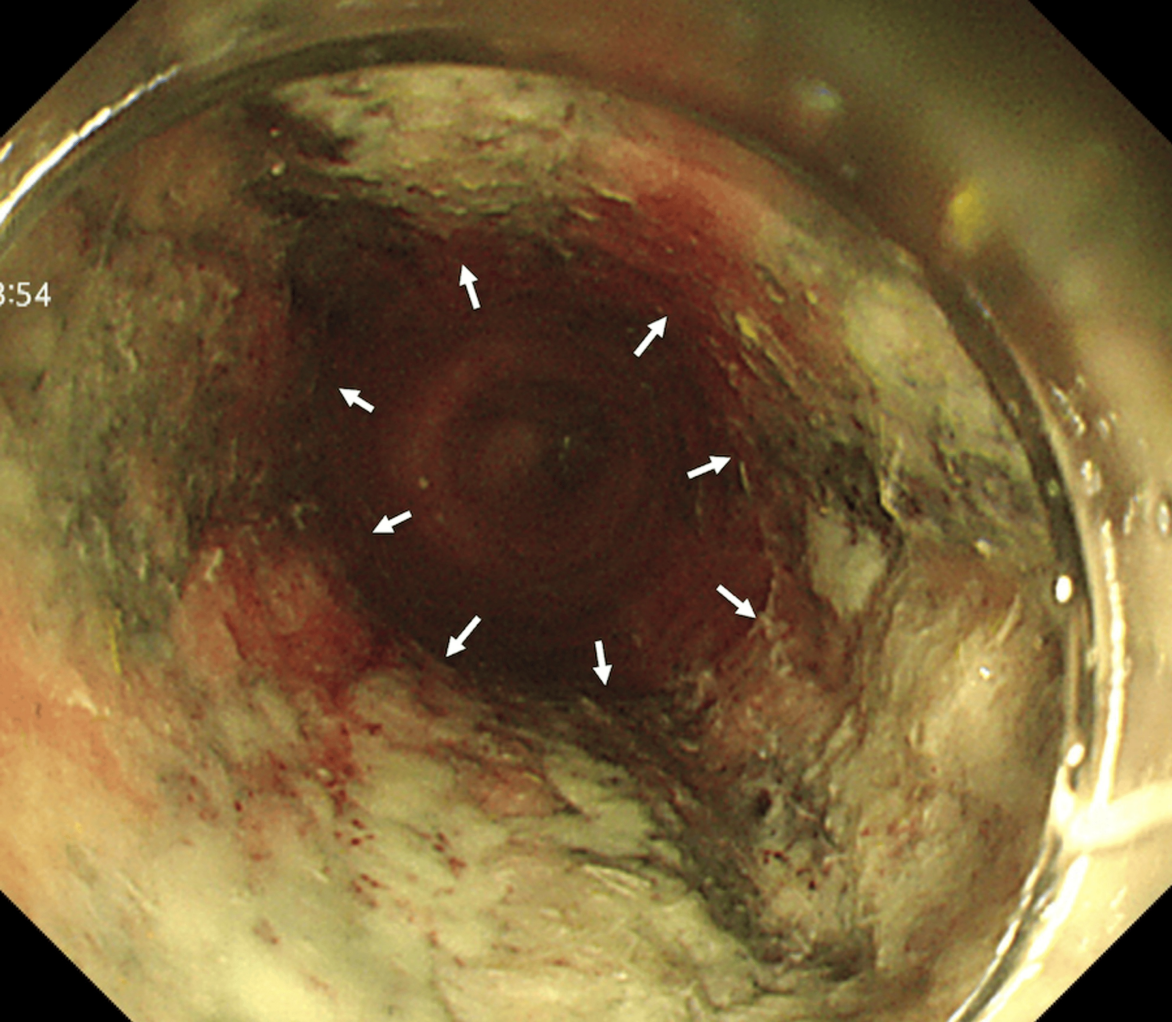
Endoscopic image following food bolus removal Endoscopic image following food bolus removal revealed circumferential esophageal mucosal edema, adherent white exudate, and black discoloration consistent with acute necrotizing esophagitis (ANE); white arrows indicate the distal (anal-side) boundary of the necrotic mucosa. The proximal (oral-side) mucosa appeared normal endoscopically but is not shown in the images.

To prevent potential gastric outlet or downstream obstruction, the bolus was transected in the stomach using a snare. Based on our experience in this case, we found that large, non-sharp food boluses are often resistant to transection using snares or endoscopic submucosal dissection (ESD) devices alone. Instead, repeated blunt fragmentation at the center of the bolus, followed by controlled folding and advancement into the stomach, appeared to be the most effective strategy for removal. Endoscopic examination revealed circumferential esophageal mucosal edema, adherent white exudate, and black discoloration, consistent with ANE (Figure 5). These changes were strictly localized to the segment corresponding to the impacted bolus; distal mucosa was normal. There was no history of caustic or thermal ingestion and no clinical evidence of infection, supporting ANE as the diagnosis in this context.

The first endoscopic attempt on hospital day 1 lasted approximately 65 minutes, mainly involving snares, nets, and forceps without significant mucosal trauma. The second attempt on hospital day 2, performed approximately 16 hours after the onset of obstruction, lasted 130 minutes using dual-channel forceps, snares, and dissection devices, but removal was unsuccessful; at that time, the esophageal mucosa showed no remarkable changes. On hospital day 3, the third procedure lasted 30 minutes and achieved removal by repeated blunt fragmentation with alligator forceps. The esophageal bolus remained impacted for approximately 44 hours from symptom onset until its removal (Table 1).

**Table 1:** Clinical timeline and endoscopic management All endoscopic procedures on hospital days 1–3 were performed under conscious sedation with midazolam. EGD: esophagogastroduodenoscopy; PPI: proton pump inhibitors

Hospital Day	Event/Findings	Endoscopic Device and Technique	Outcome
Day 1	After ingesting a large piece of pork at dinner, he developed acute dysphagia and anterior chest pain and presented to the emergency department.		Admitted for urgent endoscopic removal.
Day 1 (evening)	1st EGD: GIF-Q260J used under conscious sedation with midazolam. A large meat bolus was tightly impacted in the thoracic esophagus with no visible lumen. Attempts using grasping forceps, a retrieval net, and a snare were unsuccessful.	Initial attempts to push the bolus into the stomach were unsuccessful.	Bolus remained impacted; the patient kept NPO and was treated with intravenous PPI overnight.
Day 2 (morning)	2nd EGD: Initially, a transnasal GIF-1200N was attempted, but could not pass because of the bolus size. The scope was switched to a dual-channel GIF-2TQ260M. Two grasping forceps were introduced simultaneously through both channels for traction. A retrieval net, snare, and dissection devices (needle knife, and scissor-type knife) were also attempted.	Procedure prolonged (≈130 min). Electrosurgical transection was not possible: the fatty composition prevented effective current transmission, so mechanical fragmentation was used; thermal injury risk was considered low.	Removal unsuccessful; mucosa unchanged.
Day 3	3rd EGD: GIF-Q260J used. Repeated blunt fragmentation with alligator forceps at the bolus center achieved dislodgement and advancement into the stomach.	Shorter procedure (≈30 min).	Complete removal achieved. Circumferential edema, adherent white exudate, and black discoloration (ANE) observed proximal to impaction.
Day 4–7	Conservative management (IV fluids, NPO). WBC 14,200/μL, CRP 14.67 mg/dL on Day 4.		Day 7 EGD showed recovery with only residual white exudate.
Day 9	Oral intake resumed; patient discharged asymptomatic.		Denture fabrication recommended to prevent recurrence.

On hospital day 4, although his anterior chest pain had resolved after bolus removal, laboratory studies showed a white blood cell count of 14,200/μL and C-reactive protein of 14.67 mg/dL, indicating a marked inflammatory response (Table 2).

**Table 2:** Laboratory data on hospital day 4

Parameters	On Hospital Day 4	Reference Range
White cell count (μL)	14200	3300-8600
Neutrophils (%)	80.7	40-68
Eosinophils (%)	0.3	0-5
Lymphocytes (%)	12.3	26.0-46.6
Red blood cells (×10^4 ^μL)	524	435-555
Hemoglobin (g/dL)	15.1	13.7-16.8
Platelet count (×10^4 ^μL)	26.2	15.8-34.8
Sodium (mEq/L)	137	138-145
Potassium (mEq/L)	3.7	3.6-4.8
Chloride (mEq/L)	100	101-108
C-reactive protein (mg/dL)	14.67	< 0.3
Albumin (g/dL)	4.4	3.6-4.8
Total bilirubin (mg/dL)	1.06	0.2-1.2
Aspartate transaminase (U/L)	12	13-30
Alanine transaminase (U/L)	7	10-42
Lactate dehydrogenase (U/L)	114	135-225
Blood urea nitrogen (mg/dL)	15	8-20
Creatinine (mg/dL)	0.82	0.65-1.07

**Figure 6:**
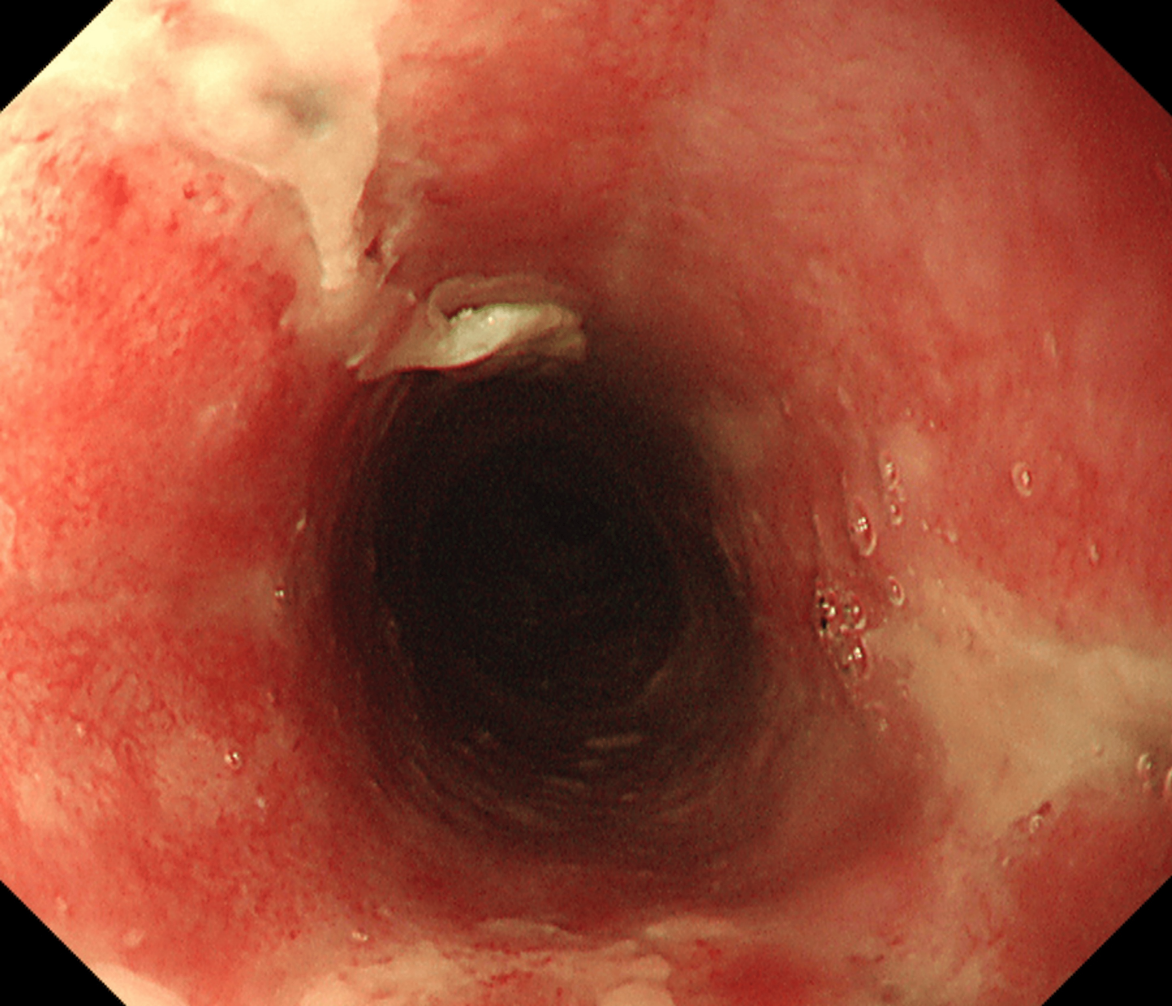
Follow-up endoscopic image on hospital day 7 Follow-up endoscopic image on hospital day 7 showed resolution of the black mucosal discoloration. Only residual white exudate remained, indicating improvement in acute necrotizing esophagitis (ANE).

Conservative management with nil per os (NPO) and intravenous fluid therapy was continued for the patient. On hospital day 7, follow-up EGD showed resolution of the black mucosal discoloration, with only residual white exudate (Figure 6).

Oral intake was resumed on the same day, and the patient remained asymptomatic. The patient was discharged on day 9. Before discharge, the patient was advised to undergo denture fabrication to improve mastication and prevent recurrence, and he agreed to the recommendation.

## Discussion

This case demonstrates an uncommon instance of food bolus impaction progressing to ANE despite the absence of typical underlying esophageal pathology. While most adult cases are associated with structural or functional abnormalities, our case highlights the potential risks of prolonged mechanical obstruction and delayed endoscopic intervention. According to the European Society of Gastrointestinal Endoscopy (ESGE) and American Society for Gastrointestinal Endoscopy (ASGE) Guideline, esophageal foreign bodies should be removed within 24 hours, as delayed removal significantly increases the risk of major complications, including perforation, retropharyngeal abscess, and aortoesophageal fistula [1,5].

Endoscopic treatment options include en bloc or piecemeal extraction using grasping forceps, alligator forceps, tripod forceps, snares, or retrieval nets, as well as the push technique, which involves gently advancing the food bolus into the stomach. However, the push technique carries a risk of perforation if excessive force is applied [1], and repeated manipulation may exacerbate mucosal damage.

ANE was first reported in 1990 and is a rare clinical condition with an estimated incidence ranging from 0.013 to 0.28% [6]. It is characterized endoscopically by circumferential black discoloration of the esophageal mucosa and histologically by mucosal necrosis [6,7]. ANE most commonly affects the distal two-thirds of the esophagus, likely due to a relatively poorer blood supply compared to the proximal third [8].

The pathogenesis of ANE remains unclear, but proposed mechanisms include ischemic injury, impaired mucosal defense, and exposure to gastric acid [7,8]. Infectious causes are extremely rare [7]. Risk factors include diabetes mellitus, infections, alcohol abuse, postoperative or malignancy-related malnutrition, and circulatory failure [7,9]. In the present patient, the absence of systemic comorbidities, the lack of clinical or laboratory evidence of infection, and the black discoloration corresponding to the site of the impacted bolus strongly suggest localized ischemia from prolonged mechanical obstruction as the primary mechanism. Diagnosis of ANE is generally based on characteristic endoscopic morphology, while histological confirmation is reserved for selected cases due to the risk of perforation or bleeding [7]. The prolonged mechanical obstruction and repeated endoscopic manipulation in our case likely contributed to local mucosal ischemia and injury, triggering the development of ANE. While repeated endoscopic manipulation could have contributed to mucosal injury, the black discoloration was localized to the segment, suggesting that prolonged obstruction was the predominant factor.

Typical presenting symptoms of ANE include hematemesis or melena; however, dysphagia, epigastric pain, nausea, vomiting, and anemia have also been reported [4]. Treatment is generally conservative, involving bowel rest, nutritional support, and gastric acid suppression. In the absence of complications, clinical and endoscopic improvement is often observed, with a reported mean time to resumption of oral intake of approximately 13 days [9]. Complications include esophageal stricture (10-20%), perforation (7%), and mediastinal abscess (6%), with a reported mortality rate of up to 32% [9]. However, death is often attributed to underlying comorbidities rather than ANE itself [9]. Most cases of ANE resolve without long-term sequelae if the acute episode improves without complications such as perforation or severe stricture [7].

In this case, a large (40 × 25 mm) meat bolus was impacted at the level of the tracheal bifurcation. Despite multiple endoscopic interventions, removal proved challenging. We speculate that sustained mucosal compression and vascular compromise ultimately led to the development of ANE. The patient recovered promptly with conservative therapy and was discharged without sequelae, likely owing to the absence of severe comorbid conditions. While the patient's favorable outcome likely reflects the absence of significant comorbidities, further studies are needed to assess whether similar outcomes can be expected in patients with ANE of different etiologies.

To our knowledge, few reports have linked prolonged food bolus impaction to subsequent ANE; this case underscores the potential for localized ischemic injury after sustained obstruction. A previous report described a case of esophageal perforation occurring within 6-8 hours after hot dog ingestion, attributed to focal transmural ischemia from direct mechanical compression [10]. In this case, a sealed obstruction may have led to a sudden increase in intraluminal pressure, contributing to mechanical rupture. However, this case did not exhibit diffuse mucosal necrosis or the black discoloration characteristic of ANE. In contrast, our case illustrates a unique progression to ANE following prolonged impaction and repeated endoscopic attempts, emphasizing the need for timely intervention and a cautious technique to minimize mucosal injury and associated complications. When endoscopic retrieval is unsuccessful or complications are suspected, escalation to surgical management should be considered in accordance with guideline-based practice.

However, this report has several limitations. First, no follow-up could be obtained after discharge, and thus, late complications such as esophageal stricture cannot be excluded. Second, high-resolution manometry was not performed, so underlying esophageal motor disorders could not be definitively ruled out. Third, because an esophageal biopsy was not obtained, EoE cannot be completely excluded [11]. Despite these limitations, the clinical course strongly suggested ANE triggered by a refractory food bolus impaction.

## Conclusions

In conclusion, this case highlights a rare but noteworthy complication of prolonged esophageal food impaction in the absence of underlying esophageal disease. Prolonged impaction (>24 h) increases the risk of ischemic injury; early extraction and structured follow-up are essential.
